# Barriers to prompt and effective malaria treatment among the poorest population in Kenya

**DOI:** 10.1186/1475-2875-9-144

**Published:** 2010-05-27

**Authors:** Jane Chuma, Vincent Okungu, Catherine Molyneux

**Affiliations:** 1 Kenya Medical Research Institute-Wellcome Trust Research Programme, P.O Box, 230, Kilifi, Kenya; 2 Centre for Tropical Medicine, Nuffield Department of Clinical Medicine, University of Oxford, UK

## Abstract

**Background:**

Prompt access to effective malaria treatment is central to the success of malaria control worldwide, but few fevers are treated with effective anti-malarials within 24 hours of symptoms onset. The last two decades saw an upsurge of initiatives to improve access to effective malaria treatment in many parts of sub-Saharan Africa. Evidence suggests that the poorest populations remain least likely to seek prompt and effective treatment, but the factors that prevent them from accessing interventions are not well understood. With plans under way to subsidize ACT heavily in Kenya and other parts of Africa, there is urgent need to identify policy actions to promote access among the poor. This paper explores access barriers to effective malaria treatment among the poorest population in four malaria endemic districts in Kenya.

**Methods:**

The study was conducted in the poorest areas of four malaria endemic districts in Kenya. Multiple data collection methods were applied including: a cross-sectional survey (n = 708 households); 24 focus group discussions; semi-structured interviews with health workers (n = 34); and patient exit interviews (n = 359).

**Results:**

Multiple factors related to affordability, acceptability and availability interact to influence access to prompt and effective treatment. Regarding affordability, about 40 percent of individuals who self-treated using shop-bought drugs and 42 percent who visited a formal health facility reported not having enough money to pay for treatment, and having to adopt coping strategies including borrowing money and getting treatment on credit in order to access care. Other factors influencing affordability were seasonality of illness and income sources, transport costs, and unofficial payments. Regarding acceptability, the major interrelated factors identified were provider patient relationship, patient expectations, beliefs on illness causation, perceived effectiveness of treatment, distrust in the quality of care and poor adherence to treatment regimes. Availability barriers identified were related to facility opening hours, organization of health care services, drug and staff shortages.

****Conclusions**:**

Ensuring that all individuals suffering from malaria have prompt access to effective treatment remains a challenge for resource constrained health systems. Policy actions to address the multiple barriers of access should be designed around access dimensions, and should include broad interventions to revitalize the public health care system. Unless additional efforts are directed towards addressing access barriers among the poor and vulnerable, malaria will remain a major cause of morbidity and mortality in sub-Saharan Africa.

## Background

Prompt access to effective malaria treatment is central to the success of malaria control worldwide. The Roll Back Malaria (RBM) partnership has set for 2010 a target of ensuring that 80 percent of those suffering from malaria have prompt access to, and are able to correctly use, affordable and appropriate treatment within 24 hours of symptoms onset [1]. Most African countries are far below these targets, with only a minority of fevers being treated promptly and effectively [2-5]. The 2008 World Malaria Report states that between 2006 and 2007, only 38 percent of fevers reported among children under five were treated with anti-malarials, and only three percent were treated with artermisinin-based combination therapy (ACT) [6], the official first-line anti-malarial for uncomplicated malaria in over forty African countries [7]. The number of fevers treated promptly and effectively prior to the policy change from monotherapies to ACT was equally low [8].

Low levels of ACT uptake can be attributed to various factors including limited availability outside the public sector, high costs, poor prescribing practices, and frequent stock outs in public health care facilities [9-11]. Various initiatives to increase access to ACT have been implemented or are in the process of implementation. The Affordable Medicines Facility-malaria (AMFm) aims to enable countries to increase the provision of affordable ACT in the public, private and non-governmental organizations through co-payment at factory gate [9]. Subsidies will be accompanied by interventions such as shop-keeper training programmes so as to increase availability and safe use of ACT in the retail sector. A pilot study of this delivery approach in Tanzania reported a dramatic increase in access to ACT [10]. Community based interventions including the distribution of drugs by community health workers have been equally successful [12-15]. Interventions targeting retailers implemented in the pre-ACT era were effective in improving home management of fevers [9,16-19], and looked cost-effective [20].

In Kenya, the malaria treatment policy was changed from sulphadoxine/pyrimethamine (SP) to ACT artemether-lumefantrine (AL) in 2006 [21]. Despite the policy change, amodiaquine remains the main anti-malarial used to self-treat fever, and is widely prescribed by health workers [22-24], even when ACT is available in health care facilities [4,25]. The various types of ACT are 10 to 15 times more expensive than other anti-malarials [26,27] and their availability is currently restricted to the public and formal private health sector. Plans are however underway to provide subsidized ACT in the private retail sector through the AMFm subsidy [28]. While these are potentially good developments for malaria control in Kenya and elsewhere, existing evidence shows that the poor benefit less from malaria control interventions than higher income groups [29], and are less likely to seek prompt effective treatment when they fall sick [30,31]. Given that reaching low income groups is essential for equity and for the success of public health interventions, it is important that the factors preventing low income groups from accessing interventions and effective treatment are better understood. This paper describes access barriers to effective malaria treatment among the poorest population in four malaria endemic districts in Kenya. The WHO guidelines on malaria treatment recommend that cases of suspected malaria are treated with a full effective treatment irrespective of a confirmatory diagnostic test [27]. In this paper, prompt effective treatment is defined as ability to correctly use appropriate anti-malarials to treat suspected or confirmed malaria within 24 hours of symptoms onset.

### What does access to malaria treatment entail?

There is no consensus on what access to health care means and how it should be measured. Existing definitions present access as a multifaceted concept, influenced by multiple interrelated factors occurring at both the supply and demand level [32-37]. Recently, Thiede *et al *[37] have developed an access framework that explores three dimensions of access namely: (1) affordability, which entails the full cost of health care vis-à-vis the ability to pay for the services; (2) acceptability, which refers to the compatibility between lay and professional health beliefs, including patient perceptions of effectiveness of treatment, and the extent to which their constructions of health and healing match health workers' understanding of these issues; and (3) availability, which goes beyond physical location of health services to include opening hours, ability and willingness of providers to serve the population as well as type, range, quality and quantity of services. Factors occurring at the different dimensions interact to influence access to health care services.

Most studies that explore access to malaria treatment focus on a single determinant of access primarily distance to health care facilities, availability of anti-malarials, or utilization of health care services. However, access to malaria treatment is far broader than these indicators, with a range of demand and supply side barriers potentially hindering people from seeking prompt effective malaria treatment. In addition to financial and physical access barriers, examples of factors influencing access include perceptions of illness causation, perceived and actual effectiveness of treatment and quality of care, provider attitudes, adherence to drug dosage, power dynamics within households, and availability of good quality information. Understanding these barriers and how they interact to influence treatment-seeking among the poor is essential for improving effective case management. In Kenya, the role of distance in access to malaria treatment and morbidity levels is well documented [38-40], but efforts to understand other barriers of access have been relatively piecemeal. This paper draws on the framework of Thiede *et al *[37] to consider the range of barriers to prompt and effective malaria treatment among the poorest populations in Kenya.

## Methods

The study was conducted in four districts in Kenya purposively selected to represent different ecology and malaria transmission patterns. Kwale district on the coast with seasonal, high intensity transmission; Bondo district on the shores of Lake Victoria with high intensity perennial malaria transmission; Gucha district representing the low seasonal transmission conditions of the Western highlands; and Makueni, a semi arid district with acutely seasonal, low transmission. There are also a wide range of research activities going on in the districts as part of monitoring Kenya's progress to the Abuja targets [41-43].

According to the 1999 population census, the total population living in the districts are 952,752 people in Gucha, 952,000 in Kwale, 238,000 in Bondo and 771,545 people in Makueni [44]. Gucha district is the most densely populated with a population density of 706 persons per square kilometre, while Kwale is the least densely populated with a population density of 60 persons per square kilometre. The population density in Bondo and Makueni is 240 and 93.8 persons per square kilometre respectively. The proportion of children aged below five years in the districts ranges from 14% in Makueni to 17.3% in Kwale. The main ethnic groups in Kwale and Bondo are the Mijikenda and the Luo respectively. The Abagusii are the main ethnic group in Gucha, while the Akamba are the main residents of Makueni. Agriculture is the main source of income in all districts. Additional information on the study districts is provided elsewhere [45,46].

### Data collection

The data presented in this paper come from a broader study that aimed to explore barriers to effective treatment and prevention among the poorest populations in Kenya. Multiple data collection methods were applied in the wider study to enhance reliability and validity. They included a cross-sectional survey (n = 708 households), focus group discussions (n = 24), semi-structured interviews with health workers in-charge of primary health facilities in the districts (n = 34) and patient exit interviews (n = 361). The results presented in this paper are mainly from the FGDS and patient exit interviews, although data from the cross-sectional survey are presented where necessary to provided quantitative data on key variables of interest.

The cross-sectional survey collected data on self-reported fevers, treatment-seeking behaviour, treatment barriers, costs and sources of money for treatment. Focus Group Discussions (FGDs) explored barriers to access in more detail, gathering in-depth information on the interaction between different factors and how they influence access to prompt and effective treatment. Interviews with health workers and patient exit interviews gathered providers and facility users' perspectives on various aspects related to the access dimensions, including facility opening hours, availability of drugs and provider-patient interaction.

Survey households were selected using multi-stage sampling. A detailed description of the sampling approach is published elsewhere [45,46], but briefly: First locations-the 2^nd ^lowest administrative unit in Kenya-were selected using poverty indicator maps generated by the Kenya National Bureau of Statistics [47]. Second, four enumeration areas (EAs) located in the two poorest quintiles in each district were randomly selected. Third, a homestead list was updated and all homesteads in the EAs were selected to take part in the study. Finally, all households in the homestead were included in the study.

A two-week recall period was used to collect data on self-reported fever (indicator for malaria) and treatment-seeking patterns. FGDs participants were selected from the same areas on the basis of age and gender. Priority was given to men and women of reproductive age because children are more vulnerable to malaria in the study settings [48]. A few FGDs included older men and women because of their important role in treatment-seeking decisions [49,50]. Participants were drawn from different parts of the village to maximize diversity. FGDs were conducted up to the point of redundancy (n = 24). All FGDs were tape recorded and notes taken for in-depth interviews. Primary health care facilities were selected purposively based on their proximity to the EAs, and semi-structured interviews were held with all available health workers. Patient exit interviews were conducted with all individuals who sought care from participating health facilities on the date of the interview.

### Data analysis

Quantitative data were double entered into Microsoft visual FoxPro (version 9.0) and transferred to STATA (version 9.2) for analysis. FGDs were transcribed and analysed manually using content analysis. Data were first analysed separately for each district and then brought together to identify cross-cutting issues. The findings were grouped together to give an indication of the overall barriers that residents living in malaria endemic areas face since key themes were generally similar across the districts despite differences in malaria endemicity. To ensure trustworthiness, two researchers independently read and re-read the transcripts to identify codes, subthemes and themes.

**Figure 1:**
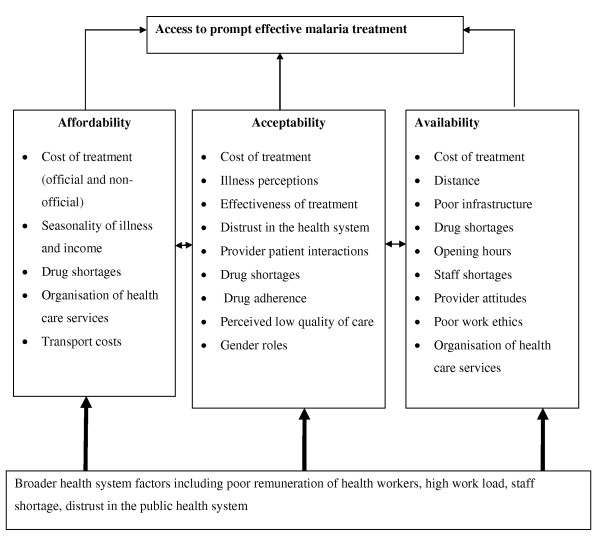
**A summary of factors influencing access to prompt effective malaria treatment**.

Findings are organized around three broad access dimensions common across various analytical frameworks [32-37]. Within each dimension, there are a number of factors affecting both demand and supply side determinants of access, although these factors do not operate independently as demonstrated in Figure 1. Quantitative data on each dimension are first presented to provide a general overview of the determinants of access at the community level, followed by qualitative data which provide more detailed insights into the access barriers, some of which are difficult to quantify.

### Ethical approval

Ethical approval was obtained from the Kenya Medical Research Institute (SSC No. 964), and the World Health Organization Research Ethics Review Committee (ID A50045).

## Results

### Self-reported fever and treatment-seeking patterns among survey households

A summary of self-reported fevers and treatment-seeking patterns is presented in Table 1. Fever was reported in 506 households (71.5%). A total of 1074 individuals (25.5%) reported fever in the two weeks preceding the survey. More fevers were reported among children under five years of age than among older children and adults (33.1% and 23.9% respectively). More than 90 percent of reported fevers were treated; treatment levels were high for both fevers reported under fives and the rest of the population (91.5% and 90.6% respectively). Most fevers were treated within the first two days of symptoms onset; 86.0% of fevers reported among children under five and 83.3% of fevers reported among the rest of the population were treated within the first two days.

**Table 1:** Treatment-seeking patterns among survey households.

Variable	Number (%) among children under five and the rest of the population		
	
	< 5 years	> 5 years	Total
Individuals reporting fever in the last 2 weeks	241 (33.1)	833 (23.9)	1074 (25.5)

Any action taken to treat fever			
• Yes	246 (91.5)	776 (90.6)	1022 (90.8)
• No	23 (8.6)	81 (9.5)	104 (9.2)

Time taken before taking first treatment action			
• < = 2days	191 (86.0)	636 (83.3)	827 (83.9)
• > = 3 days	31 (14.0)	124 (16.2)	155 (15.7)
• Don't know	4 (0.5)	0 (0)	4 (0.41)

Distribution of actions taken in response to illness			
• Self treatment			
◦ Drugs from shops	146 (31.9)	534 (35.9)	388 (37.7)
◦ Drugs from chemists	70 (15.3)	175 (17.0)	245 (16.5)
◦ Herbs	25 (5.5)	76 (7.4)	101 (6.8)
• Public health facility	134 (29.3)	197 (19.2)	331 (22.2)
• Private health facility	23 (5)	59 (5.8)	82 (5.5)
• Faith based facility	18 (3.9)	30 (2.9)	48 (3.2)
• Mobile clinic	26 (5.7)	78 (7.6)	104 (7.0)
• Other	15 (3.3)	28 (2.7)	43 (2.9)

Reasons for not seeking treatment			
• Lack of money	13 (56.5)	51(63.0)	64 (61.5)
• Illness not serious	11 (47.8)	32 (39.5)	43 (41.3)
• Other	3 (13.0)	12 (14.8)	15 (14.4)

The informal sector was the main source of treatment for all age groups. About 32% of fevers reported among children under fives were treated using drugs bought from the shops, 15.3% were treated using drugs bought from chemists and 5.5% were treated using herbs. Among adults, 37.7% of reported fevers were treated using drugs from the shops, 17.0% using drugs from the chemist and 7.4% were treated using herbs. Only a minority of fevers were treated at public and private health care facilities (39.0%). The proportion of fevers treated in a public health facility was 29.3% for illnesses reported among children under five, and 19.2% for illnesses reported by older children and adults. Private health facilities were hardly used, with only 8.7% of all reported fevers treated at either a private or faith-based facility. Children aged below five years were more likely to receive treatment at a formal health care facility than other age groups (p = 0.001).

### Barriers to malaria treatment

#### Affordability related factors

Affordability is also commonly known as financial access [33,34,37]. The cross-sectional survey identified lack of money as a major barrier for seeking treatment: 61.5% of individuals who did not seek treatment reported that cash shortage was the main barrier (Table 1). Individuals who treated fevers were asked if they had enough money to pay for treatment. In 40.0% of all treatment actions taken, money was not readily available at the time of seeking treatment (Table 2). Lack of cash was common among both individuals self-treating using drugs from the shops (38.9%), and those that sought treatment in the formal sector (41.8%). Where cash was not readily available households adopted different coping strategies (Table 2). Borrowing was the main source of cash for treatment (25.4%). Money was mainly borrowed from friends and neighbors (38.5%), and from relatives (36.9%). Other coping strategies included being treated on credit at a health facility (17.6%), gifts (10.1%) and sale of assets (10.0%). Sale of labor was hardly used as a source of money to pay for treatment, but it was the main source of money for paying debts that arose due to the treatment-seeking (41.3%). Assets were also frequently sold in order to pay for illness related debts (41.3%).

**Table 2:** Sources of money to pay for treatment.

Variable	n = 1022 (%)
Had enough money to pay for treatment	
• Yes	613 (60.0)
• No	409 (40.0)

Sources of money to pay for treatment	
• Cash savings	636 (62.2*)
• Borrowing	260 (25.4)
• Gifts	103 (10.1)
• Casual labour	34 (3.3)
• Sale of assets	102 (10.0)
• Credit at health facility	180 (17.6)
• Waivers	12 (1.2)
• Other	26 (2.5)

Money borrowed/credit paid back at the time of survey?	
• Yes	166 (37.7)
• No	274 (62.7)

Sources of cash to clear debts	
• Cash	15 (9.0)
• Casual labour	69 (41.3)
• Sale of assets	69 (41.3)
• Gifts	12 (7.2)
• Other	12 (7.2)

*Total percentage > 100 because multiple strategies were sometimes adopted

Qualitative data also identified affordability as a major barrier to access. Affordability related barriers reported in the FGDs included:

##### Cost of treatment

Costs of treatment as a barrier to access was a predominant theme in all FGDs. High costs reportedly prevented people from seeking effective treatment despite knowing that malaria should be treated with appropriate anti-malarials. Women experienced particular problems raising money for treatment. Alternative cheap and ineffective sources of treatment were often sought:

*"There is this drug called coartem which is very expensive *[referring to the first-line anti-malarial in Kenya]. *Its price is about 900-1200 Kenya shillings (KES). When you get a prescription to buy such a drug, given our economic situation, one cannot afford to buy it, even when you are seriously ill. That is why we have to resort to other means like panadol *[pain reliever] *if only to get relief. We are even more disadvantaged as women because our sources of income are limited compared to men's." *(FGD, Female)

*"Sometimes parents do not have money to take their children to a health facility. We buy medicines like panadol *[pain reliever] *which we use to bring the fever down. Then we believe that the child has recovered, but that is not true...malaria continues to gain strength *[meaning becomes severe]." (FGD, Male)

Study participants reported that people who lacked money to pay for treatment at the government facilities were not given appropriate anti-malarials. This impacted on people's perceptions of quality of care, acceptability of services and contributed to poor drug adherence:

*"We do not get proper treatment because money is required at the dispensary.., but if you do not have the money, you cannot be treated. If you have less money, then you will be given drugs that cannot cure you." *(FGD, Female)

*"If you do not have enough money, they *[health workers] *give you a one-day dose and ask you to go with money the next day to collect the remaining dose...But where is the money? They give us an under-dose which is not good" *(FGD, Male)

Other affordability issues reported included cost of transport and waiting time:

*"You can find a household with four ill children. Giving them treatment becomes difficult. First, transporting them to the facility is a problem, raising money to pay for treatment is difficult, and when you get to the dispensary, you wait the whole day." *(FGD, Male)

##### Seasonality of illness and income sources

Seasonality is an important factor influencing treatment-seeking for malaria. In all FGDs, seasonality of malaria transmission and income sources were identified as barriers to access. FGD participants reported that malaria mainly occurs during the peak agricultural season when people are busy in the farms, and when most make a large proportion of their annual cash income. Being ill during this period therefore had significant impacts on people's income and sometimes led to failure to treat illness in order to continue with income generating activities. Also, the queues at the health facilities were reportedly longer than in other times of the year, and drug supplies were felt to be inadequately adjusted to meet the high demand in the peak season:

*"There are constant stock-outs during the rainy season because there are many ill people. We suggest that the quantity of drugs supplied during the rainy season be increased to match the rising number of illnesses." *(FGD, Male)

*"Getting proper treatment is usually a problem during the wet season because many people fall sick. The dispensaries are also full during this period." *(FGD, Male)

*"Sometimes you are sick but you just have to continue working because you have children looking upon you for food. If you do not work, you will not harvest or get any money to buy food for the children." *(FGD, Female)

Incomes were also reported to be seasonal and unreliable, and most people struggled to make ends meet during the difficult months of the year. During this period, seeking effective treatment was beyond households' budgets:

*"The months from October to February are very dry and most of us have nothing to do to raise money. You can look after someone's goat for a day to get KES 20, but that is nothing for anyone who has a family to feed, let alone take a child to a health facility" *(FGD, Male)

*"There are particular months when it is difficult to get money. During this time, there is no rainfall and therefore no farming activities going on...there is no water for our vegetable farms.., there is no money"*. (FGD, Female)

##### Informal or 'under-the-table' payments

In addition to formal charges, people occasionally reported giving health workers some money to enable them receive 'good' treatment quickly. These informal charges were sometimes high, increased the cost of treatment, and acted as an access barrier:

"S*ome of them ask for tea *[meaning under-the-counter payment] *of about KES 50 or KES 100 if one is to get good drugs and faster treatment." *(FGD, Male)

#### Acceptability related factors

Acceptability is sometimes referred to as cultural access [32]. It involves the interactions between the health care system and service users, provider and patient attitudes, and expectations of each other [32,51]. It also includes the compatibility between lay and professional health beliefs, patients' perceptions of effectiveness of treatment, and the extent to which their constructions of health and healing match health workers' understanding of these issues [32]. The cross-sectional survey collected data on individuals' satisfaction with the quality of services received at formal health care providers as an indicator of acceptability. The results indicated that 311 individuals (55.0%) who sought treatment from formal health care providers were satisfied with the services; 209 (36.9%) were dissatisfied and; 45 (8.0%) were indifferent. The main reasons given for the dissatisfaction were the quality of drugs received (23.2%) and lack of drugs (23.2%); low confidence in staff ability to offer good quality treatment (17.8%), and lack of diagnostic tests (10.1%). Satisfaction with services was linked primarily to availability and effectiveness of drugs, confidence in staff, and short waiting time (69.1%, 13.1% and 3.6% respectively).

Qualitative data provided more insights on acceptability related determinants of access:

##### Provider-patient interactions and perceptions on health workers' attitudes

Provider and patient attributes, expectations, beliefs and perceptions were identified as key factors influencing acceptability of formal health care services. In Bondo district, older clients reportedly found it difficult to accept treatment by youthful providers, associating young health workers with inadequate training and poor quality of care, including disrespectful behaviour. In Gucha and Kwale districts there were similar concerns about community health workers (CHWs). Several comments suggested that these concerns might be linked to broader problems of distrust in health workers and dissatisfaction with the replacement of older more familiar staff:

*"It is their qualifications that we doubt.... The young boys are not good but the older providers who worked here before were good. We feel the drugs are there but they do not know how to diagnose diseases and administer appropriate medication. They do not know how to hold the syringe; they hold it like it is some pen for writing" *(FGD, Female).

*"We have these people who are CHWs but they give treatment at the health facility. When you go there, they are the ones to inject you and when you get concerned and ask him 'we have just come with you from home, are you qualified to treat me?' He tells you to stay there untreated. And when you go to the qualified doctors to complain, they say that if you think the CHWs are not qualified, you should bring your own doctor to the dispensary. At this time you keep quiet and know that you are not going to get proper treatment." *(FGD, Female)

In many FGDs across the four districts, health workers were reported to be inconsiderate and uncaring, and these negative opinions apparently impacted on people's willingness to seek care at public facilities. Although in some cases health workers were said to lack commitment and not to have the patients' interests at heart, others felt the health workers were not solely to blame; that their attitudes resulted from broader health system factors associated with high work load and low remuneration:

*"The other thing is the attitude of the health workers. They do not care about the patients. It is like they do not have a human heart or blood. They sit doing nothing all day...we have to plead with them to attend to us." *(FGD, Male)

*"It is because *[referring to why quality of care is low] *the staff are few and the overwhelming number of patients makes it impossible for them to give due attention to each patient. They are also paid very low salaries yet they are trained...they should be paid well*." (FGD, Female)

*"We cannot blame him *[the provider] *because he does his best. Not even leaving for tea break or lunch...he treats non-stop" *(FGD, Male)

##### Perceptions of illness causes, effectiveness of treatment, and distrust in quality of care

Malaria was reported to be a common problem in all FGDs across the four districts. The causes of malaria were not always clear, with participants in about half the FGDs in all districts attributing it to dirty water, coldness or weak blood. High awareness of the need to treat malaria in formal facilities was demonstrated in all FGDs. While people did not always use anti-malarials to treat fevers, they attributed this to affordability and availability barriers rather than lack of awareness of appropriate drugs. People reported that they could easily diagnose malaria but that their diagnoses sometimes conflicted with those of health workers. Even when blood tests were reported as negative, patients reported that they sometimes bought malaria drugs from shops or chemists anyway. In such cases this may mean that additional, possibly unnecessary, costs are incurred by households:

*"I was told that I did not have malaria, but when I went to the chemist and bought malaria drugs for KES 80, I got better. The following day I could attend to my duties without much difficulty." *(FGD, Male)

*"Sometimes you feel that you are suffering from malaria but when you visit the hospital, the tests show that there is no malaria in your body. You are given panadol which cannot cure you. If you have money, you will go to buy malaria drugs." *(FGD, Male)

##### Poor adherence to treatment

Poor adherence to treatment emerged as a barrier to effective treatment. Where drugs were available and issued to patients at health facilities, it was reported that people did not always adhere to the dosage, either to save the medicine for future use, or because drug administration timings were inconvenient, especially for mothers who left their children behind to go and work on the farms or to look for casual labor:

*"Some parents do not follow the instructions they are given by providers. Many parents fail to administer the full dose to children as soon as they notice signs of recovery. This happens mainly for the syrups...mothers want to keep them for another day *[meaning another illness episode]." (FGD, Male)

*"It is the commitments that mothers have that make them forget to give their children medicine. When the child feels better, her mind switches to other things such as the ever pressing need to cultivate vegetables by the lake. Once she is by the lake she will not return home to give the child medicine." *(FGD, Female)

Unavailability of services and desperation were other factors that contributed to poor adherence. People reported that patients failed to complete the dosage, either because the medicine was not available at the facility, or the health workers did not open the dispensary due to other commitments:

*"Treatment can be effective, but for it to be effective one has to comply. But when the health worker recommends, for example three injections, the patient ends up receiving one because the provider is either absent or the medicine is not available." *(FGD, Male)

*"At times your neighbor might give you some drugs that they are not using, and you take those drugs not knowing what they treat or whether they are expired." *(FGD, Female)

#### Availability related factors

Availability (or physical access) refers to whether health services and providers are supplied in the right place at the right time, and whether the services offered correspond with population's needs [32,33,35,37]. Key availability themes that emerged were facility opening hours, drug shortages, and location and organization of health services.

##### Facility opening hours

One out of the 34 facilities covered was reported to operate 24 hours, seven days a week. Most opened at 8.00 am every day (n = 28), and closed at 4.30 pm (n = 19). In 20 facilities, health workers reported being contactable for emergency services out of hours through mobile phones. In 10 facilities, the in-charge reported having had to close the facility, either to attend a workshop (n = 3), when summoned to the district headquarters by the district medical officer for health (n = 2), or during annual leave (n = 3). These facilities were operated by only one health worker and, consequently, there was no one to offer services in their absence.

The limited operating hours were repeatedly reported as barriers to access in all districts, especially during the weekends when primary health care facilities remained closed:

*"Sometimes the illness starts in the evening or during the weekend when the dispensary is closed, so we are forced to buy drugs from the shops. If it is a serious illness, you suffer throughout the weekend or go to the district hospital if you have the money, but this is rare. Few people have money to go to the district hospital." *(FGD, Male)

Closely related to official opening hours is that facilities apparently often do not open on time. Community members attributed this primarily to poor work ethics among the health workers and inaccessible roads during the wet season:

*"The provider comes to the facility at his own time; he opens late everyday and closes early. There is no consistent time of opening or closing the facility and over the weekends there is no one to treat us." *(FGD, Male)

*"The provider leaves early because there is only one vehicle plying this route and if he misses it, there are no other means to town. During the rains, the roads are impassable and at times the dispensary nurse-in-charge cannot get here" *(FGD, Female)

Others felt that health workers did the best they could under the circumstances. Some facilities were understaffed, some had only one health worker, and in their absence the health facilities remained closed or were operated by community health workers.

*"One problem is that we have only one health worker who leaves on Friday early afternoon and reports back to work on Monday afternoon. So from Friday through to Monday we do not have anyone to treat us. Sometimes he goes for a seminar the whole week and during this period the dispensary remains closed" *(FGD, Female)

##### Drug shortages

Drug availability is another key factor influencing access to treatment. Individuals who sought treatment from public health facilities in the two weeks preceding the survey were asked if they received all the prescribed drugs from the hospital pharmacy and whether or not they were issued with a prescription to buy drugs from a private chemist. The results indicated that 95 (30.0%) of people who visited public health facilities did not get drugs from the hospital pharmacy and were issued with a prescription to buy drugs elsewhere. Of these, only 31 (32.8%) individuals bought the prescribed drugs. Among exit interview participants, 140 (38.8%) did not receive drugs from the facility because they were out of stock.

Persistent shortages of anti-malarials in public health care facilities discouraged people from seeking effective treatment. Participants in all FGDs were very vocal about chronic drug shortages in the public health facilities and the implications this had on affordability and treatment-seeking behaviour:

*"When you take your child to the health facility...first you queue and pay for registration, then you are sent to the first room where you pay for the rubber stamp, after that you are told to go to the laboratory, where again they tell you to pay for the tests, then you get to the pharmacy and you are told there are no drugs. You go through this long process and use all the money, but all you have are receipts. What are you going to do with receipts? They should have left you with your money to buy panadol or to go to another health facility." *(FGD, Female)

*"The problem is that when you go to the hospital, the health worker says there are no drugs, so all he does is to write prescriptions or refer you to the district hospital. If you have no money for drugs, where will you get money to go to the district hospital? That is why malaria is killing us...You will suffer until God decides to take the illness away." *(FGD, Male)

There were strong perceptions that health workers' diverted public drugs to private chemists and clinics where they often referred people to buy the prescribed medicines. This behaviour, it was reported, was responsible for the chronic drug shortages in public health care facilities:

*"The health workers at the dispensary often ask patients to go to their homes later in the evening or during the weekend for treatment because there is no medicine at the dispensary but they use drugs they have taken from the dispensary." *(FGD, Male)

*"He opened a drug shop at the market and would steal our drugs to sell to us at his shop until he was transferred..." *(FGD, Female)

Shortage of anti-malarials in the health facilities was also attributed to Coartem- the 1^st ^line drug in Kenya- being expensive and, therefore, the government could not afford to restock the drug on time:

*"The drugs at the dispensary are not good. We do not get well after taking them. I think it is because the price of the new drug *[referring to ACT] *is very high, and so it is never in stock. The malaria drug that is sometimes in stock is fansidar *[SP], *which no longer cures malaria." *(FGD, Male)

While many apparently blamed the health workers, a few pointed out that it was difficult to confirm whether the problem was in the supply of drugs from the government or the behaviour of the health workers. Making health facilities more accountable to the community was identified as a potential mechanism for reducing and/or confirming suspicions of drug theft:

*"It is difficult to verify these claims. Nobody knows whether the government brings drugs and no one checks the pockets of the health workers to confirm that they steal drugs." *(FGD, Female)

*"The community should have access to records showing the amount of drugs received and how they have been dispensed. This way, we can know if they *[health workers] *steal drugs or not." *(FGD, Male)

##### Location of health facilities

Physical location of health facilities in relation to service users and availability of transport influence where, when and what sort of treatment is sought. In almost all FGDs across the four districts, participants reported that they had to travel for long distances to a health facility. The poor road network and limited sources of transport further compounded the problem. Long distances also involved extra time and financial costs:

*"There is only one vehicle that passes here at 6 am and at 3 pm to and from town respectively and when one misses it, then one has to wait till the following day.... We have nothing to do in case of an emergency" *(FGD, Male)

*"It is the distance from health facilities that makes it difficult for us to seek good quality treatment fast. It is not easy to get a vehicle to the facilities and meet the costs of transportation as well as for the treatment at the same time. Sometimes we use bicycles but it is too far" *(FGD, Female)

##### Organization of health care services

The way health care facilities are structured may promote or hinder use of health care services and thus act as a barrier to effective malaria treatment. Seeking treatment from public health facilities was reportedly an unnecessarily long and frustrating process, involving a number of stages. Patients typically: pay the registration or card fees; take the card for stamping and sometimes pay a stamping fee; see the health worker who might recommend a parasitaemia test; go to the laboratory and then return to the health worker with the results; and finally go to the pharmacy to receive available prescribed drugs. Each stage can involve significant queuing and delays, making the system cumbersome, with negative implications for patients trust in the providers' and in the health system.

## Discussion

This paper applies an access framework to explore access barriers to effective malaria treatment among the poorest populations in Kenya. Here, the results are discussed in more detail and compared to the wider literature on access to health care. Access to malaria treatment is clearly influenced by multiple factors occurring at both the supply and the demand level. A summary of the interrelated barriers to access identified in this paper is provided in Figure 1.

Overall, a positive finding was that most reported fevers were treated within the first 24 hours of symptoms onset. Like in many parts of sub-Saharan Africa, over 50% of reported fevers were treated using drugs bought in the informal sector, with only about 20% treated in the public sector, the main source of formal care in the study settings. In Kenya, Coartem, the first-line anti-malarial, is not sold in the informal sector, implying that a high proportion of fevers treated in this sector were not treated effectively. Moreover, evidence suggests that even fevers treated in the formal public sector in the recent past have hardly been treated using the recommended anti-malarials [3,4,24,52,53], due to various reasons related to all access dimensions including drug shortages, patient's preferences and perceived high costs of drugs [25]. Additional data on the types of medicines used to treat malaria in the era of ACT, and the level of adherence to drug regimes would be useful in measuring the country's progress towards the Abuja targets.

### Demand-side factors

Affordability is a major barrier to malaria treatment and health care throughout sub-Saharan Africa. On the demand side, health care charges, seasonal incomes, transport costs and waiting time all interact to make affordability a major barrier for the poorest households. Ready cash to pay for treatment was hardly available and households often mobilized additional resources mainly through borrowing from their social networks. Lack of money contributed to poor drug adherence since health workers and drug shops were reported to readily issue incomplete dosages. It was also reported that people who lacked money to pay for treatment were given inappropriate drugs at government facilities because they were reportedly cheaper than the recommended anti-malarials. Poor prescribing practices and drug adherence has potential implications for drug resistance and acceptability of services. For example, drug effectiveness was one of the factors that patients attributed to the satisfaction or otherwise of health services. Incomplete doses and less effective drugs are likely to clear the symptoms in the short-term, with the possibility of a recurring episode. As shown by the findings, recurring illnesses that are treated with incomplete doses or ineffective drugs can erode the trust in the health system, and thus impacting negatively on acceptability of health services.

All public health facilities in Kenya charge user fees. In an attempt to make malaria treatment affordable, the Kenyan government provides free anti-malarials to all public health care facilities, which ideally should be dispensed free of charge to malaria patients. Malaria treatment is therefore, officially free to all Kenyans. In a further development, in 2004, registration fees for malaria patients in all primary health care facilities were eliminated. However, the findings presented in this paper and elsewhere suggest that health care charges remain a significant barrier to access and that the 'free treatment' policy is not fully implemented for various reasons including [45]: (1) poor policy design, where patients are required to pay consultation fees before being seen by a health worker; (2) low revenue, especially in districts where malaria is the main illness. Exempting malaria patients from paying fees in malaria endemic districts impacts heavily on the amount of revenue collected; (3) the difficulties of identifying patients suffering from malaria since many illness conditions have symptoms similar to malaria and many primary health care facilities do not have laboratories and; (4) shortage of drugs supplied by the government meant that facilities had to raise additional money through charging fees in order to raise money to purchase drugs. The government should work closely with health workers, district health management teams and health facility committees to ensure that people do not pay for anti-malarials in the public health sector. Non-health related interventions that address insecurity and fragility of income sources can have a significant role in minimizing affordability barriers and improving effective treatment [54].

Regarding acceptability of treatment, about half of the people that sought care from formal health services reported that they were satisfied with the quality of services. Availability and effectiveness of drugs were the main factors given for satisfaction with health services. However, many community members in the study setting appeared to have strong negative perceptions of health workers attitudes and their ability to provide good quality care. Health workers were often reported to be disrespectful to patients and their qualifications were sometimes questioned. Such perceptions may often relate to cultural beliefs, which affect provider-patient interaction, but also demonstrate the reluctance to accept new health workers following transfer of more familiar and trusted staff. The fact that the negative perceptions towards health workers' age, gender and qualifications were expressed mainly by elderly women implies that they may have considerable cultural concerns, firstly, in exposing their bodies for injection to providers who were predominantly young males, and secondly, in tolerating the language of providers, which they regard as disrespectful to their age and social status as married women. In districts where CHWs assisted health workers to provide services at the facility level, community members expressed concerns regarding their expertise, and they were reluctant to be attended by CHWs who were mainly from the same community and well known to them. These findings demonstrate the challenges of using CHWs to support health workers in resource constraints regions. Plans are under way in Kenya to roll out the community health strategy that heavily relies on resources at the lower level, including CHWs, to improve the health status of the population. The success of this strategy will to some extent depend on the degree of CHWs acceptability at the community level.

Although the impact of negative perceptions of access to effective malaria treatment is difficult to measure, treatment-seeking behaviour has been shown to be strongly influenced by social relationships. People draw heavily on their social networks for advice; advice that is shaped by perceptions and rumors about health workers, quality of care and the health system in general [55-57]. Gilson notes that provider-patient relationships are influenced by patients' attitudes towards providers [58]. For example, providers personally known to patients or of the same ethnic group or gender may be more trusted. However, providers may also introduce or reinforce negative patient perceptions through their own practices [58]. Health system constraints, such as poor remuneration, high workload and staff shortages, also play an important role in influencing provider-patient relationships [51].

Although the role of provider patient relationships in hindering or promoting access is well documented [59-61], interventions to improve the situation are inadequately considered in policy design, particularly in low income countries. In high and middle income countries, mechanisms such as patients' rights charters exist to provide information to service users and improve on provider-patients relationships [62,63]. Similar initiatives have been introduced in Kenya but the extent to which the needs of the poor and vulnerable populations are met has not been documented. Building trust in the public health system through improving quality of care, making providers more accountable to service users, and listening to community voices are essential to promoting acceptability. Such interventions require long-term planning but a potential starting point should be a clear commitment and willingness by policy makers to work towards promoting and revitalizing the public health system.

### Supply side factors

On the supply side, the main barriers to access included facility opening hours, distance to health care facilities, poor road networks, drugs and staff shortages. About a third of individuals who sought care from public health facilities did not get drugs from the hospital pharmacy because they were out of stock. Over half of the people issued with prescriptions to buy medicines outside the hospital pharmacy did not buy them.

People were particularly concerned about the chronic drug shortages in public facilities, shortages that were mainly attributed to inappropriate use by health workers. Participants also reported that these shortages were more serious during the wet season when the number of malaria cases was high and health facilities could not cope with the increased demand in malaria treatment. Drug supplies were not adjusted to sustain the increased demand. Lack of drugs can impact on acceptability of health services. When people do not receive the appropriate drugs due to frequent stock out, they develop negative perceptions of health workers and have limited trust in the health system. Drug shortages also contribute to high costs of treatment, and thus impact on affordability. Drug shortages in public health facilities mean that people have to buy drugs from private chemists often at a higher price, despite already having incurred some charges - for example consultation and laboratory fees - at the facility. It is worth considering the organization of health care services to ensure that people only incur charges at the health services if drugs are available.

Shortage of first-line anti-malarials has been reported as a main problem in the Kenyan health system [11,24,45,53] and elsewhere in SSA [3,52]. A recent study conducted in Kenya revealed that [11]: 26 percent of public health facilities studied did not have any of the four AL weight-specific packs in stocks and 75 percent had at least one weight specific pack out of stock; a large proportion did not have packs for the youngest ages (the most vulnerable group to malaria); and some facilities reported not having ACT for close to two months. It is not clear why public health facilities experience chronic drug shortages, but it is stipulated that the drug supply system in Kenya is very inefficient. Other reasons include seasonality of illness, failure to account for differences in district health needs when designing the drug kits and delayed supply [45]. The Kenyan government is aware of the drug shortage problems in public health facilities and plans are under way to change the drug supply system from one where facilities receive standard drug kits, to a pull system where facilities order their drugs based on their need. The new approach has been piloted in some parts of the country, although there is no information on its effectiveness. It is important that the new drug supply system is evaluated to ensure that it contributes to improved drug supply in the public health care system.

Distance to health facilities and poor road networks was not only a barrier to the community seeking care, but they also influenced the presence of health workers at the health facility. The public health facilities discussed in this study are located in some of the remotest areas in the districts with no housing facilities for the health workers. Consequently, many of the health workers resided in neighbouring towns and they often used public means of transport on a daily basis, transport that was heavily unreliable, and hardly operated during the rainy season due to the poor road conditions. Few studies have documented the impact of physical infrastructure on health workers ability to provide good quality services. While it is difficult to confirm whether provider lateness was mainly due to poor road network and unreliable transport, the findings demonstrate the relationship between availability and acceptability. Community members attributed lateness of providers and few working hours to poor work ethics and limited interest in their work and rarely associated it with some of the constraints faced by health workers.

Primary health care facilities were the main sources of care in the study settings. These facilities only operated within certain hours and remained closed during the weekend. Beyond the official opening hours, there was no source of formal care in these settings. Geographical location of health care facilities has been reported as a barrier to malaria treatment in Kenya [40,42,64], but few studies identify primary health care facilities opening hours as a barrier to effective treatment. The findings have shown that primary health care facilities remain closed during the weekends, and other periods when the sole health worker is not available due to official or personal commitments. Yet these facilities are the closest to the population and are the only formal sources of treatment for the poorest population. Policy makers should reconsider the operating hours of primary health care facilities in remote rural settings. Other interventions that can address the availability barriers include providing mobile clinics and outreach programmes. These interventions will, however, require that additional health workers are deployed to these facilities and that appropriate anti-malarials and other essential drugs are readily available.

Table 3 summarizes the possible policy actions to addresses barriers to effective malaria treatment identified in this paper. These actions involve the three access dimensions and include both short-term and long-term interventions. Interventions relating to the broader health system are also identified.

**Table 3:** Policy actions to address barriers of access to prompt and effective malaria treatment.

Short to medium term interventions			
**Affordability factors**	**Acceptability factors**	**Availability factors**	**Broader health system factors**

• Provide ACTs through the retail sector • Re-enforce the free malaria treatment policy in public health facilities • Educate people on the free malaria treatment policy	• Improve communication skills for health workers • Educate community on the importance of drug adherence • Strengthen transparency and accountability of health services to the community (e.g. making information on resources readily accessible to the community through their representatives)	• Extend operating hours for dispensaries and health centres to include weekends • Improve management skills for health workers and health facility committees • Ensure sustained supply of anti-malarials, adjust supply for seasonality • Provide housing facilities for health workers working at primary care level in remote rural areas	• Recruit more health workers in primary care facilities • Take services close to the people through mobile clinics and outreach programmes • Improve overall availability of drugs and other supplies

**Long term interventions**			

• Remove user fees in public health facilities • Provide social protection for the poor and vulnerable through strengthening livelihoods	• Build culturally sensitive health systems (educate health workers on need to respect clients and be sensitive to their needs) • Empower clients to demand good health services through information and communication at grass root level	• Build additional primary health care facilities in remote areas • Increase the number of health workers in rural remote areas to at least two health workers per facility	• Address the problem of health workers shortage • Improve organization and administration of service, for example through packaging services in way that minimizes waiting time • Improve the organization and working culture of the public health system

### Limitations

A limitation of this work is that the findings are based on comments and reports of samples of people living in four poor rural settings in Kenya. Although the aim was to understand barriers to access among the poorest population, the urban poor might face different barriers due to contextual differences related to better infrastructure and more health care facilities. Regarding formal treatment, people discussed public health facilities far more than private facilities. This is most likely because no private providers operated in close proximity to these low income settings. However, the fact that people did not discuss about treatment in the private formal sector suggests that this sector is beyond the reach of the poorest population.

## Conclusions

Ensuring that all individuals suffering from malaria have prompt access to effective treatment is a major challenge for resource constrained health systems. Policy actions to address the multiple barriers of access should be designed around all dimensions of access, and should also include broad interventions to revitalize the public health care system. Unless additional efforts are directed towards addressing the barriers to access among the poor and vulnerable population, malaria will remain a major cause of morbidity and mortality in SSA.

## Competing interests

The authors declare that they have no competing interests.

## Authors' contributions

JC and CM were involved in the conception and design of the study. JC and VO participated in data collection, analysis and write up. JC wrote the first draft; all authors commented on drafts, read and approved the final manuscript.
